# Endoscopic Removal and Conservative Treatment of a Small Bowel Perforation Caused by a Toothpick: A Case Report

**DOI:** 10.7759/cureus.57254

**Published:** 2024-03-30

**Authors:** Asuka Watanabe, Dai Nakamatsu, Tsutomu Nishida, Yoshifumi Fujii, Naoto Osugi, Kengo Matsumoto, Masashi Yamamoto, Koji Fukui

**Affiliations:** 1 Gastroenterology, Toyonaka Municipal Hospital, Toyonaka, JPN

**Keywords:** colonic diverticulitis, foreign body ingestion complications, endoscopic removal, toothpick, small bowel perforation

## Abstract

This case report describes a unique instance of small bowel perforation in a 49-year-old woman caused by an ingested toothpick. Initially suspected of colonic diverticulitis, a final diagnosis of small bowel perforation was made later, and the toothpick was successfully removed via endoscopy. This case emphasizes the need to consider foreign body ingestion in the differential diagnosis of abdominal pain and demonstrates the feasibility of conservative endoscopic approaches in similar cases.

## Introduction

Foreign body ingestion is a relatively common occurrence, resulting in an object being spontaneously expelled by the body. The probability of complications such as gastrointestinal tract perforation is less than 1% [1,2]. However, in rare instances, ingestion can lead to serious outcomes such as shock or fistula formation between the foreign body and blood vessels, leading to a poor prognosis [3]. Several factors contribute to the ingestion of foreign bodies, including swallowing difficulties, older age, the influence of alcohol or drugs, diminished consciousness, eating quickly or while talking, the nature of food, and the use of dentures or medical devices. Sometimes, an individual swallows a foreign object, such as toothpicks, without realizing it.

Small bowel perforations are critical medical emergencies that typically result from trauma, inflammatory diseases, or medical procedures. Swallowing foreign objects, such as toothpicks, is uncommon but can lead to significant challenges in diagnosis and treatment. Toothpicks can cause severe gastrointestinal problems, including perforation. However, these cases are rarely reported because they are infrequent, and early symptoms are often unclear.

This case is notable for small bowel perforation caused by an unconsciously swallowed toothpick. This highlights the need for imaging in patients with unexplained abdominal pain. Despite unclear initial symptoms, a computed tomography (CT) scan showed a perforation by a long, sharp foreign object, leading to appropriate treatment.

This case report underscores that gastrointestinal perforation caused by a small foreign object, such as a toothpick, can be effectively treated with endoscopy, with surgery also considered a treatment option.

## Case presentation

A 49-year-old healthy woman presented at a clinic with acute pain on the left side of her abdomen that had persisted since the day before November 2022. Sharp and intermittent pain was not related to eating. The patient's medical history was unremarkable. An elevated white blood cell count (14,700/μL) and c-reactive protein (CRP) (9.04 mg/dL), combined with physical findings, indicated possible acute colonic diverticulitis. She was treated with intravenous antibiotics (sulbactam sodium) for seven days, but her symptoms persisted. On the 12th day, she was referred to our hospital for further examination. Blood tests showed a reduced white blood cell count (8,100/μL and CRP level (3.35 mg/dL) (Table 1).

**Table 1:** Laboratory data on admission

Parameters	On admission	Reference range
White cell count (/μL)	8100	3300-8600
Neutrophils (%)	76.3	40-68
Eosinophils (%)	0.5	0-5.0
Lymphocytes (%)	17.1	26.0-46.6
Red blood cells (×10^4^/μL)	442	389-492
Hemoglobin (g/dL)	13.2	11.6-14.8
Hematocrit (%)	40.8	35.1-44.4
Platelet count (×10^4^/μL)	27.5	15.8-34.8
Prothrombin time (%)	100	70-130
Activated partial thromboplastin time (sec)	32	24-34
D-dimer (μg/mL)	1.2	< 1.0
Sodium (mEq/L)	136	138-145
Potassium (mEq/L)	4.2	3.6-4.8
Chloride (mEq/L)	99	101-108
Fasting Glucose (mg/dL)	99	73-109
C-reactive protein (mg/dL)	3.35	< 0.3
Albumin (g/dL)	4.0	4.1-5.1
Total bilirubin (mg/dL)	0.39	0.2-1.2
Direct bilirubin (U/L)	0.12	0-0.4
Aspartate transaminase (U/L)	16	13-30
Alanine transaminase (U/L)	13	7-23
Lactate dehydrogenase (U/L)	170	135-214
Alkaline phosphatase (U/L)	70	35-104
γ-Glutamyltranspeptidase (U/L)	26	9-32
Creatine kinase (U/I)	62	41-153
Blood urea nitrogen (mg/dL)	11	8-20
Creatinine (mg/dL)	0.57	0.46-0.79

**Figure 1:**
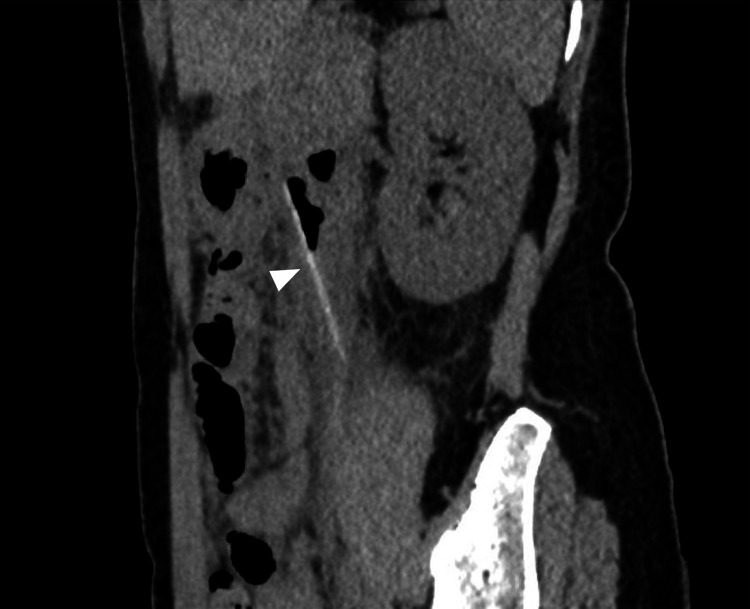
Abdominal contrast-enhanced computed tomography An abdominal contrast-enhanced computed tomography scan showed a 6 cm linear opacity in the jejunum, indicative of foreign body penetration near the ligament of Treitz without free air. The arrow also indicates the foreign body.

Her body temperature was 37.1°C, her blood pressure was 126/72 mmHg, and her pulse rate was 63 beats per minute. She was 172.5 cm tall and weighed 50.8 kg. Physical examination revealed a flat and soft abdomen with mild spontaneous pain and tenderness on the left side, but no signs of peritoneal irritation or intestinal obstruction were observed. There was no history of alcohol or tobacco use. Her medical history included surgery for ovarian cysts and a cesarean section, but no comorbidities, including psychiatric disorders or use of internal medications. Abdominal contrast-enhanced computed tomography (CT) revealed a 6 cm linear object in the jejunum, suggesting a foreign body piercing the small bowel wall near the ligament of Treitz (Figure 1).

**Figure 2:**
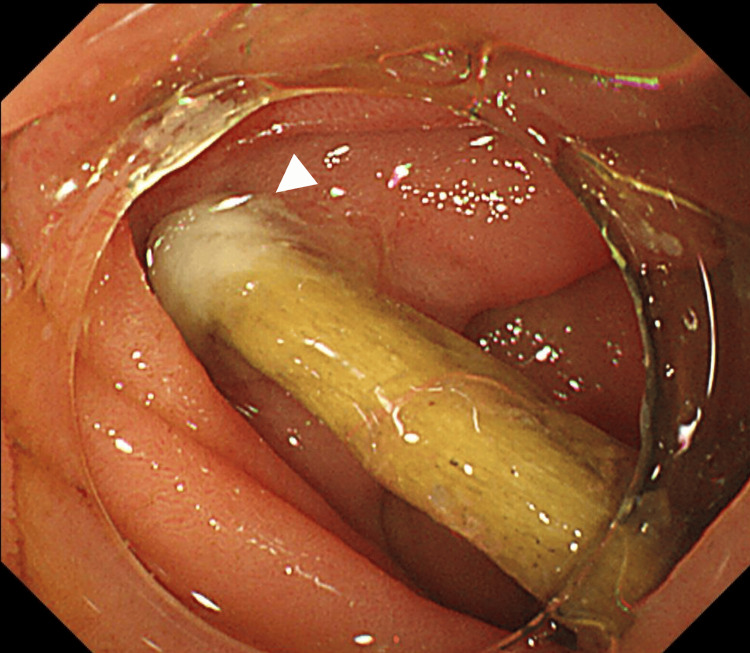
Endoscopic examination Endoscopic examination revealed a rod-shaped foreign body penetrating the small bowel wall near the ligament of Treitz. The arrow indicates the penetration site.

Considering the improvement in symptoms and CT findings, we decided to endoscopically remove the foreign body. We continued fasting and antibiotics because the inflammation was mild, peritonitis was localized, and the object was long and thin with a small penetration [4,5]. The next day, we used a small-caliber colonoscope (PCF-Q260JI, Olympus Optical Co., Tokyo, Japan) with a transparent hood to begin the examination. Fluoroscopy revealed a rod-shaped foreign body penetrating the small bowel wall near the ligament of Treitz (Figure 2).

**Figure 3:**
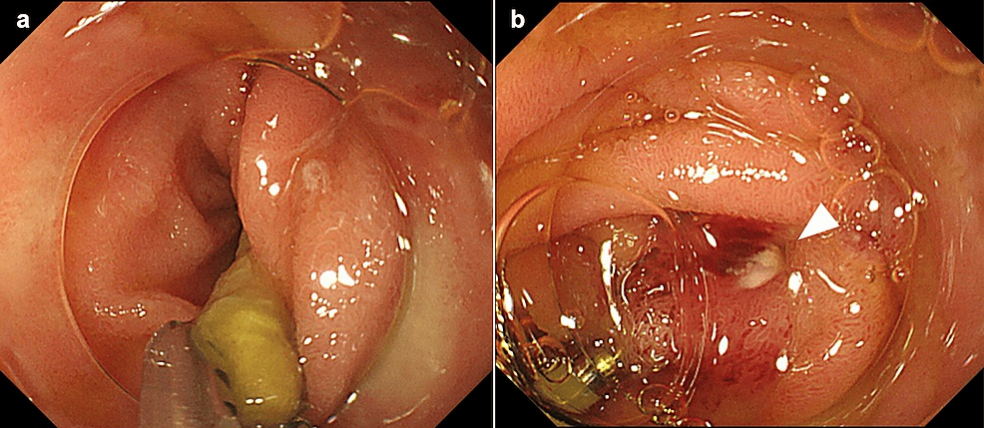
Endoscopic examination The visible end of the foreign body in the lumen was carefully grasped using crocodile forceps and pulled into the cap (a), enabling successful endoscopic removal without complications (b). The arrow indicates the penetration hole.

**Figure 4:**
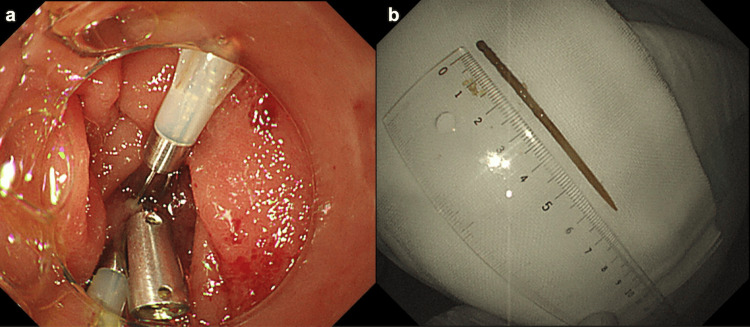
Endoscopic examination Endoscopic examination confirmed that the perforation site had been sutured using a clip (a). The removed object was identified as a 7 cm toothpick (b).

The end of the object in the lumen was grasped with forceps (Rat Tooth Alligator Jaw Grasping Forceps®; Olympus Optical Co., Tokyo, Japan) and pulled into the transparent cap attached to the endoscope tip (Figure 3a), successfully removing it endoscopically without complications (Figure 3b). The perforation was sutured using an endoscopic clip (Figure 4a). The removed object was a 7 cm toothpick (Figure 4b).

**Figure 5:**
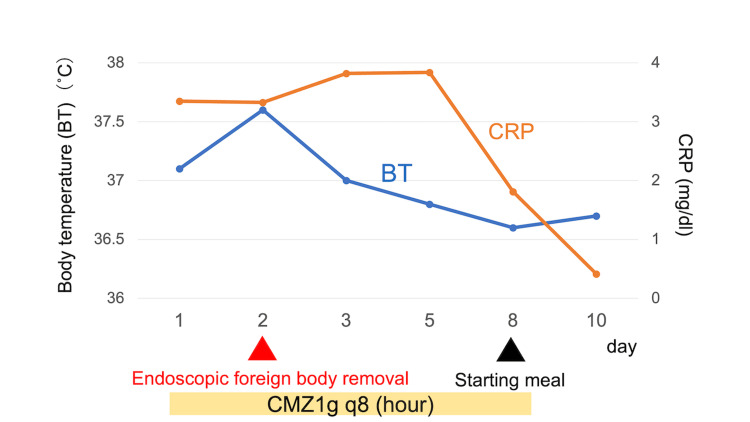
Clinical Course CMZ: cefmetazole

The removed object was a 7 cm toothpick (Figure 4b). In Japan, a typical toothpick is 6-7 cm long and is used for removing items caught between the teeth or in place of a bite-sized skewer for fried or grilled foods. After the treatment, we asked the patient about the episode of swallowing the toothpick, but she did not recall it. The patient started drinking water without worsening pain or inflammation. The patient was managed with antibiotics and monitored, and her condition improved. She began eating six days after removing the toothpick and was discharged on day 10 (Figure 5).

## Discussion

This case highlights a rare instance of small bowel perforation caused by a swallowed toothpick, initially suspected to be colonic diverticulitis based on the symptoms. Ingesting foreign objects that lead to bowel perforation is uncommon, occurring in less than 1% of cases [1,2]. Chicken bones are common in the United States [6], and fish bones are the most common objects in Japan [7], with toothpicks being less common [8]. Many patients are unaware they have swallowed a toothpick [9], making the diagnosis challenging. Symptoms are generally common and cause delays in diagnosis and management. The reported locations of toothpicks at removal include the esophagus (2%), stomach (20%), duodenum (23%), small intestine (18%), and large intestine (37%) [9]. The absence of specific symptoms often leads to a delayed diagnosis. While half of the cases require surgery, endoscopic removal works in 19-30% of cases [3,9]. The European Society of Gastrointestinal Endoscopy guidelines for removing foreign bodies from the upper gastrointestinal tract in adults suggest that CT scans are helpful not only for detecting foreign objects but also for identifying complications such as abscesses [10]. The visualization of toothpicks on CT may depend on the type of wood, and some may be easier to detect without intravenous contrast [11].

Oku et al. immersed toothpicks in water and examined the Hounsfield unit (HU) values of CT from 24 hours to 120 hours (5 days) after immersion [12]. The HU values of CT increased from the second day, peaked on the third day, and remained unchanged for the next five days. In this case, 12 days had passed between the initial onset of symptoms and the CT evaluation; therefore, it is necessary to recognize the possibility that the toothpick, which had absorbed digestive juices, could be recognized as a highly absorbent area on CT and could not be recognized immediately after aspiration.

In such cases, deciding whether to perform endoscopic removal is crucial. Although surgery is usually needed to remove foreign small-bowel objects, endoscopy can reduce surgical risks. This successful endoscopic extraction underscores the value of considering less invasive methods in similar cases. Surgery is usually the treatment of choice for intestinal perforation caused by foreign objects [8]. However, in cases with a small perforation or penetration and the possibility of endoscopic closure without complications such as peritonitis, abscess, or damage to nearby organs, or in patients at high risk due to comorbidities or advanced age, endoscopic removal and wound closure using techniques such as clips or over-the-scope clip (OTSC) can be considered [13,14].

Furthermore, this case emphasizes the importance of suspecting foreign body ingestion when diagnosing abdominal pain, especially when the initial assessments are inconclusive. A high level of suspicion and careful examination are critical for timely and effective management.

## Conclusions

This report provides evidence that endoscopic management of foreign-body-induced small bowel perforations can be a viable and safe alternative to surgery in suitable cases. However, each case should be assessed individually, considering the patient's condition and the nature of the foreign object.
